# From lab reagent to metabolite: the riboswitch ligand guanidine as a relevant compound in bacterial physiology

**DOI:** 10.1128/jb.00073-25

**Published:** 2025-05-22

**Authors:** Payton Bowman, Hubert Salvail

**Affiliations:** 1 Division of Immunity and Pathogenesis, Burnett School of Biomedical Sciences, University of Central Florida College of Medicine 124507, Orlando, Florida, USA; The Ohio State University, Columbus, Ohio, USA

**Keywords:** guanidine, guanidinium, riboswitch, nitrogen metabolism, stress response, efflux pump

## Abstract

Efforts of the last 20 years in validating novel riboswitches led to the identification of numerous new motifs recognizing compounds with well-established biological functions. However, the recent characterization of widespread classes of riboswitches binding the nitrogen-rich compound guanidine raised questions regarding its physiological significance that has so far remained elusive. Recent findings established that certain bacterial species assimilate guanidine as a nitrogen source via guanidine-specific enzymes and transporters and that complete ammonium oxidizers can use it as a sole source of energy, reductant, and nitrogen. The frequent association of guanidine riboswitches with genes encoding guanidine efflux transporters also hints that bacteria may experience the burden of guanidine as a stressor during their lifestyle. A major gap in understanding the biology of guanidine resides in its natural source. While metabolic pathways responsible for guanidine synthesis were defined in plants, only a few guanidine-producing enzymes have been identified in bacteria, despite indications that the model organism *E. coli* may produce guanidine. This review summarizes how riboswitch research unveiled guanidine as an important compound in living organisms and the recent findings advancing our knowledge of guanidine biology. We also highlight open questions that will orient future research aiming at gaining further insights into the biological relevance of guanidine.

## INTRODUCTION

More than two decades ago, the first naturally occurring mRNA elements regulating gene expression by directly binding target ligands, termed riboswitches, were discovered in bacteria (1–4). Since then, more than 55 classes of riboswitches have been characterized (5, 6), advancing our general knowledge of gene regulation and stress response, but also unveiling unexpected biological roles for compounds whose relevance had remained elusive. A notable example is the discovery of riboswitches recognizing c-di-AMP and c-AMP-GMP (7, 8), which hinted at important biological functions for the two molecules that were later revealed to act as second messengers in key cellular processes such as biofilm formation, chemotaxis, sporulation, and virulence (9–11).

The recent identification of four distinct classes of guanidine riboswitches, widespread in bacteria (12–16), raised the possibility that guanidine serves important cellular functions beyond its well-known laboratory application as a protein denaturant (17, 18). Many of the genes controlled by these riboswitches encode guanidine-specific efflux transporters expressed in the presence of guanidine (19), thereby suggesting that bacteria experience guanidine stress in the niches they occupy. That riboswitch-regulated genes involved in guanidine import and assimilation are found in bacterial species capable of utilizing guanidine as a sole nitrogen source (20, 21) also highlights guanidine’s possible role as a relevant metabolite in nitrogen-poor environments. While guanidine-producing enzymes were previously identified in bacteria and plants (22–26), the biological function of internally produced guanidine remains somewhat elusive. Here, we review recent findings in guanidine metabolism and stress response and discuss the major knowledge gaps in our understanding of guanidine biology.

## RIBOSWITCH VALIDATION REVEALED THE BIOLOGICAL RELEVANCE OF GUANIDINE

**Fig 1:**
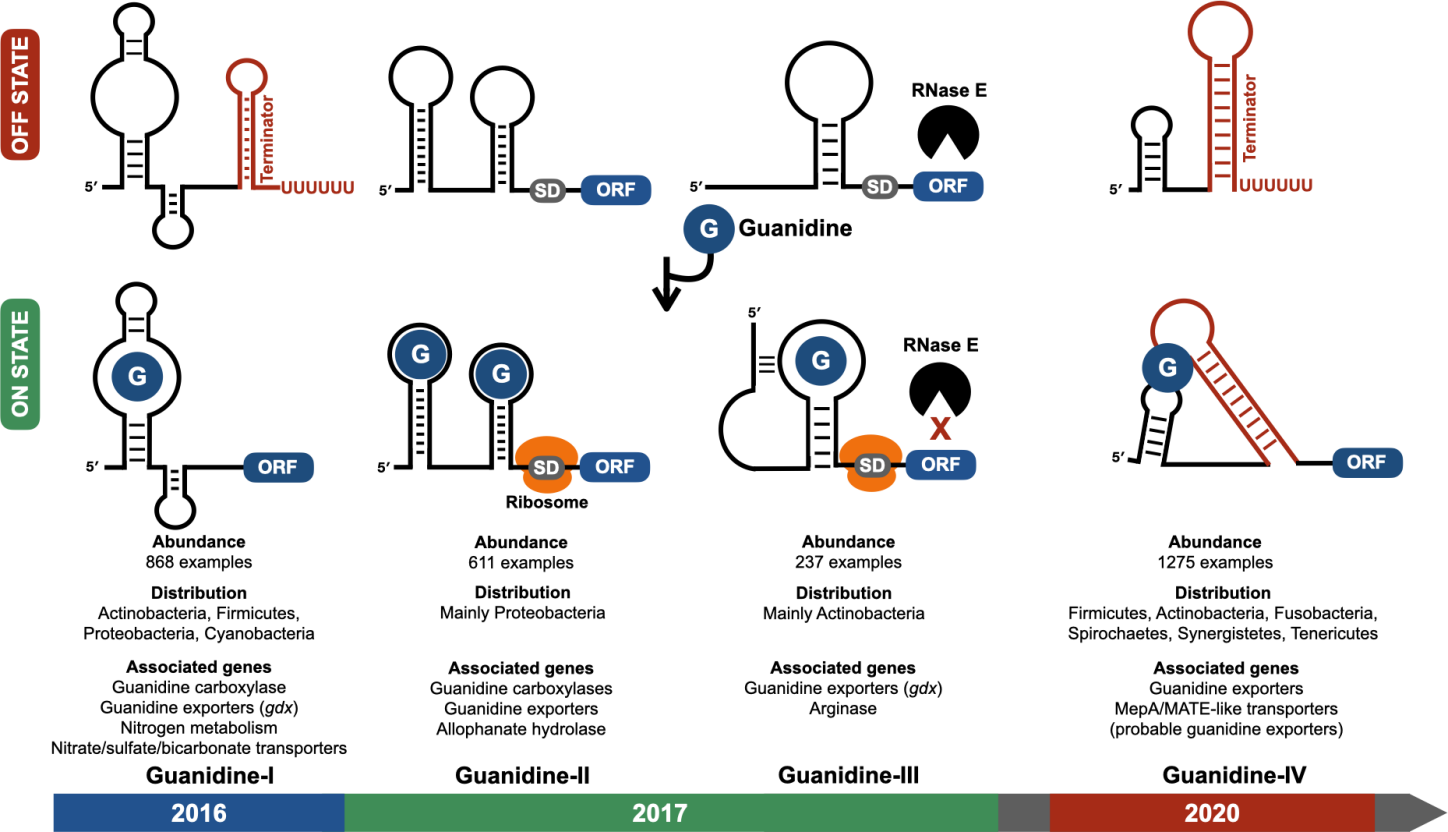
The four classes of guanidine riboswitches. The upper part of the figure depicts the gene regulation mechanism of each class with the RNA conformation adopted in the absence (OFF STATE) or in the presence (ON STATE) of guanidine. The abundance, distribution, and most frequent gene association for each class are indicated at the bottom, as well as their year of discovery. Guanidine-I riboswitch usually operates as a transcriptional “ON switch” in which guanidine binding prevents the formation of a terminator structure, thus resulting in transcription elongation into the downstream ORF. Guanidine-II functions as a translational switch using two distinct aptamers cooperatively binding guanidine to induce structural changes promoting translation initiation. Guanidine-III controls RNA stability and translation of the associated ORF, with guanidine binding promoting the formation of a pseudoknot, causing transcript stabilization by preventing RNase E from scanning the mRNA in search of cleavage sites and freeing the Shine–Dalgarno sequence for the ribosome to initiate translation. Guanidine-IV acts as a transcriptional switch, with guanidine binding stabilizing a pseudoknot structure, allowing transcription elongation into the downstream open reading frame by preventing the formation of a terminator structure. SD, Shine–Dalgarno. ORF, open reading frame.

Guanidine was first described in 1861 as a thermal decomposition product of the nucleobase guanine (27). Since then, guanidine and its derivatives have been exploited in a range of applications, including the use of nitroguanidine for the production of explosives (28), aminoguanidine for the manufacture of silk and wool dyes (29), and guanidine hydrochloride for protein denaturation in routine laboratory work (17, 18). The utilization of guanidine for industrial purposes was also accompanied by a growing interest in microbes capable of degrading guanidine as potential bioremediation agents for cleaning up contaminated wastewater generated from the manufacture of guanidine-derived products (30, 31). Although guanidine has been detected in plants (32) and suggested to be produced in humans through the cleavage of canavanine (33), its biological significance was not recognized until the discovery of the first guanidine riboswitch in 2016 (13) (Fig. 1).

In 2004, a bioinformatic effort to find new riboswitch classes identified the RNA element *ykkC* as a widespread riboswitch candidate with 868 examples in *Actinobacteria*, *Firmicutes*, *Proteobacteria*, and *Cyanobacteria* (34, 35). The lack of apparent biological connection among the genes associated with *ykkC* and their misannotation complicated the identification of the cognate ligand. Several of these genes were annotated as encoding urea carboxylases that were later revealed to prefer guanidine over urea as substrate (13). Another common class of genes associated with the *ykkC* motif was annotated as encoding small multidrug resistance (SMR) transporters, which are promiscuous exporters of antibiotics (34). Those were later shown to actually encode efflux transporters that are highly selective for guanidine (19). Because of the challenge of inferring *ykkC*’s ligand from its gene association, an unbiased approach was used in which ~2000 growth conditions with hundreds of compounds were screened for the activation of a reporter construct of *Bacillus subtilis*’ *ykkC* motif representative fused to the beta-galactosidase (*lacZ*) gene (13, 36). Only guanidine addition triggered the activation of the *ykkC-lacZ* reporter (13), therefore suggesting *ykkC* to be a guanidine riboswitch. This was then confirmed by *in vitro* and *in vivo* demonstrations of guanidine selectively binding *ykkC* and inducing structural changes that promote transcription of the genes associated with the riboswitch (13), as well as the crystal structure providing the structural basis for guanidine recognition by *ykkC* (37). The *ykkC* motif was then named “guanidine-I” (Fig. 1).

The identification of guanidine as *ykkC* (guanidine-I)’s cognate ligand prompted the reevaluation of the function of its associated genes. Since genes annotated as encoding urea carboxylases were found to often be under the control of guanidine-I riboswitches, questions on the physiological relevance of this regulation were raised. Because urea carboxylases and urease were presumed to be redundant in promoting urea decomposition and that contrary to most urea carboxylase genes, urease genes are not riboswitch-controlled, guanidine was hypothesized to be the actual substrate of urea carboxylases. Indeed, guanidine was demonstrated to be the preferred substrate of urea carboxylases over urea (13), with ~75% of urea carboxylase genes, most of them being riboswitch-controlled, predicted to encode enzymes having guanidine as substrate, and the rest, which are not riboswitch-associated, predicted to encode urea-specific enzymes (38). The difference of a single nucleotide in the active site of the enzymes seems to dictate the substrate specificity, with aspartate being found in guanidine carboxylases and asparagine in urea carboxylases (21). The SMR proteins encoded by the guanidine-I-regulated genes seemed to belong to a different clade than the ones previously shown to efflux a wide range of compounds and antibiotics (39, 40), thus suggesting they may transport guanidine. In agreement with this hypothesis, SMR protein genes controlled by guanidine-I were shown to encode selective guanidine exporters (13) that were later renamed Gdx (guanidine exporter) (19).

The validation of guanidine-I was also accompanied by *in vivo* data suggesting that guanidine is endogenously produced in bacteria. This was based on the observation that a guanidine-I riboswitch gene reporter displays increased activity in an *E. coli tolC* mutant strain as compared to the wild-type strain in minimal medium, and that a compound with mass-to-charge ratio and retention time similar to those of guanidine was detected by high-resolution liquid chromatography mass spectrometry (LC-MS) under these conditions (13). Because TolC exports a wide range of small toxic molecules (41), possibly including guanidine, it was suggested that high intracellular levels of guanidine accumulate in the absence of *tolC*, thus leading to the activation of the guanidine-I reporter. Further investigation is necessary to confirm whether guanidine is produced by *E. coli* and exported via TolC.

The fact that proteins highly selective for guanidine have their expression controlled by a guanidine-sensing RNA, together with data suggesting guanidine to be produced by *E. coli,* highlighted the potential biological significance of guanidine. This was further supported by the validation of three additional classes of guanidine riboswitches that followed the identification of guanidine-I.

Shortly after the identification of guanidine-I, two other guanidine riboswitches, initially named mini-*ykkC* and *ykkC-III*, and then renamed guanidine-II and guanidine-III (Fig. 1), respectively, were characterized (15, 16). Both guanidine-II and guanidine-III are associated with genes encoding efflux proteins and metabolic enzymes, similar to those whose expression is controlled by guanidine-I. The two riboswitch classes differ in their abundance and distribution, with guanidine-II having 611 examples, found mostly in Proteobacteria, such as *E. coli*, *Salmonella enterica,* and *Klebsiella pneumoniae*, and guanidine-III having 237 unique representatives, mainly found in *Actinobacteria* (35). Unlike guanidine-I, which was demonstrated to regulate the transcription of its associated genes (13), guanidine-II and guanidine-III were predicted to function as translational switches in which guanidine binding makes the Shine–Dalgarno sequence more accessible for the ribosome to initiate translation (15, 16) (Fig. 1). Guanidine-III also controls mRNA stability, with guanidine promoting the formation of a pseudoknot preventing RNase E from scanning the mRNA in search of cleavage sites, thereby protecting it from degradation (42).

Few years after the successive identification of guanidine-I, -II, and -III, two independent studies simultaneously reported the existence of a fourth class of guanidine riboswitch (Fig. 1), further hinting at the biological importance of guanidine in bacterial lifestyle (12, 14). Guanidine-IV was previously defined as the *mepA* motif because of its frequent association with *mepA* genes encoding MepA/MATE-like proteins annotated as multidrug efflux proteins. These proteins likely function to expel guanidine from cells (14), based on the previous demonstration that most of the efflux transporter genes controlled by guanidine riboswitches encode proteins highly selective for guanidine (19). The rest of the genes under the control of guanidine-IV are commonly associated with the other classes of guanidine riboswitch, many of them encoding EmrE-like proteins that probably export guanidine (12, 14). Guanidine-IV is an abundant riboswitch class with 1,275 distinct representatives found across six phyla (12, 14). It operates as a transcriptional switch via an unconventional architecture that differs from the canonical riboswitch design (Fig. 1). Unlike the classic arrangement in which the aptamer region partially overlaps with the expression platform to allow gene regulation upon ligand binding (5), guanidine-IV’s aptamer consists of two distant loops, with one of them residing in the stem of a transcription terminator. Guanidine binding promotes the formation of a pseudoknot bridging the two loops, which prevents the terminator structure from forming, thus allowing transcription elongation into the associated open reading frame (12, 14).

The wide distribution of four abundant classes of guanidine riboswitches controlling the expression of proteins specialized in guanidine efflux and degradation strongly suggested guanidine to be physiologically relevant in bacterial lifestyle. This then motivated further work to better understand the biological role of this intriguing nitrogen-rich molecule.

## BACTERIA USE GUANIDINE-SPECIFIC DETOXIFICATION SYSTEMS

**Fig 2:**
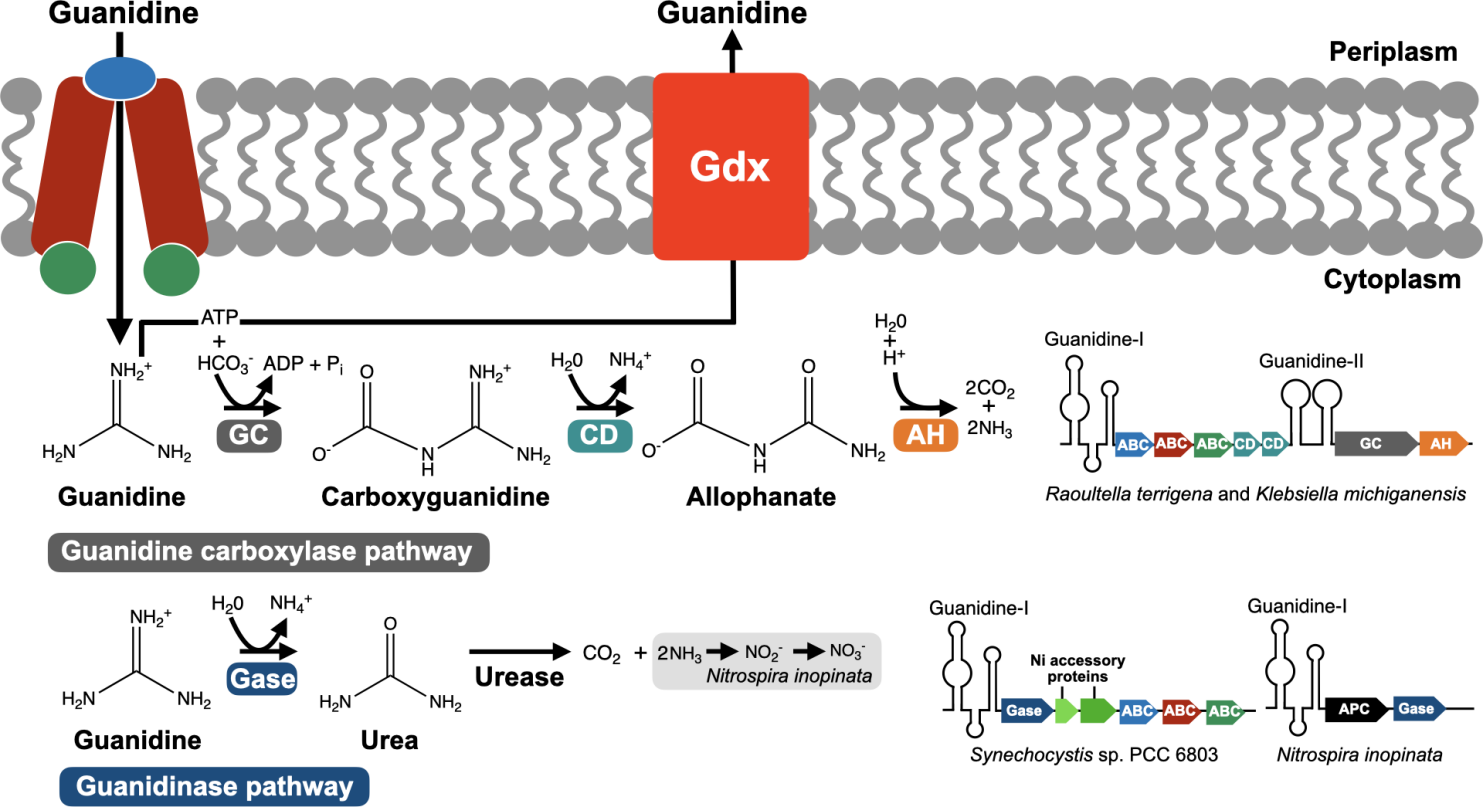
Guanidine degradation and export pathways. After importing guanidine via ABC transporters, the bacterial species *Raoultella terrigena* and *Klebsiella michiganensis* use the guanidine carboxylase pathway to degrade guanidine. *Synechocystis* sp. PCC 6803 and *Nitrospira inopinata* rely on the guanidinase pathway, with the former species importing guanidine via an ABC transporter and the latter most likely through an APC superfamily permease. *N. inopinata* is a comammox that can oxidize ammonia generated from guanidine degradation to nitrite, and then to nitrate through nitrification. Most bacterial species with guanidine riboswitches, including *Raoultella terrigena* and *Klebsiella michiganensis*, can detoxify guanidine using the Gdx exporter. The genes involved in guanidine degradation and import form operons that are under the control of guanidine-I or guanidine-II riboswitches. *Synechocystis* sp. PCC 6803’s guanidinase is encoded in the same operon as two accessory proteins loading Ni^2+^ ions into the active site of the enzyme. GC, guanidine carboxylase; CD, carboxyguanidine deaminase; AH, allophanate hydrolase; Gase, guanidinase; ABC, ABC transporter; APC, APC superfamily permease.

The discovery of four classes of guanidine riboswitches unveiled the only guanidine-specific stress response known to date in bacteria, which consists in the expression of guanidine efflux transporters in response to increased intracellular levels of guanidine (Fig. 2) (19, 43). This is most likely to mitigate the toxic effect of guanidine externally acquired or endogenously generated through metabolic activity. Given the chaotropic properties of guanidine (17), its intracellular accumulation or binding to cell surface may cause protein denaturation and misfolding. This is consistent with the induction of the heat shock response upon treatment of *E. coli* cells with guanidine, probably resulting from the accumulation of misfolded proteins (44).

Many of the genes controlled by guanidine riboswitches are annotated as *emrE* (13–16). EmrE is one of the most well-studied SMR transporters and acts as a multidrug efflux protein with broad substrate specificity. It has been shown to protect bacteria from a wide range of toxic compounds and antibiotics, including ethidium, methyl viologen, tetracycline, and streptomycin (45–47). Before the validation of the first classes of guanidine riboswitches, several bacterial genomes were known to encode proteins highly similar to EmrE, but that are not expelling drugs (48). This was, for example, the case of the SugE protein in *E. coli* that shares 30% sequence identity and 60% similarity with EmrE (49). The function of the protein had remained elusive partly due to its initial mischaracterization as a suppressor of *groEL* mutation (the gene name *sugE* stands for “Suppressor of GroEL”) (50). The plasmid phenotypically complementing the *groEL* mutation harbored an extra sequence upstream of *sugE*, including the *ecnB* gene encoding entericidin B. Given that *sugE* alone failed to complement a *groEL* strain when overexpressed (51), it is possible that *ecnB* is the actual suppressor of the mutation.

The apparent lack of sequence signature distinguishing SMR proteins exporting drugs, such as EmrE, from those that are not, like SugE, has made the prediction of their function challenging. The discovery that several SMRs have their expression under the control of a guanidine riboswitch suggested they could transport guanidine (13, 15, 16). Sequence alignments and phylogenetic analysis revealed that the experimentally validated multidrug exporters clustered in a single minority protein clade distinct from the clades comprising SMR proteins controlled by guanidine-I, -II, and -III riboswitches (19). SMR proteins can either form homodimers when encoded by a single gene or heterodimers when specified by adjacent genes that have arisen through gene duplication (19). They localize in the inner membrane with four transmembrane domains. A series of experiments assessing radiolabeled guanidine transport by purified riboswitch-regulated SMRs reconstituted on proteoliposomes established that, unlike EmrE drug exporter, they function as selective guanidine exporters operating as antiporters coupling the export of one molecule of guanidine with the import of two protons (19). These SMRs were then renamed after their function as Gdx for guanidinium export (guanidine occurs as the protonated guanidinium cation under physiological conditions) (Fig. 2). As guanidine export was revealed to be the major function of SMR proteins, with only a few of them promoting multidrug resistance, it was hypothesized that multidrug exporters may have recently evolved from ancestral Gdx proteins in response to recent stressors (19). This would then suggest guanidine to be an ancient stressor for bacteria.

While the vast majority of riboswitch-associated SMR genes are chromosomally encoded, drug-exporting SMRs are typically found on plasmids or associated with transposons, which is indicative of their recent dissemination through horizontal gene transfer (48). However, *gdx* genes under the control of guanidine-II riboswitch have been found on multidrug resistance plasmids of several Gram-negative bacilli species (e.g., *E. coli* and *Salmonella enterica*) isolated from contaminated foods and retail animals (52–54). Plasmid-encoded Gdx proteins are highly similar in sequence to those chromosomally encoded, and both were shown to confer bacterial resistance to various quaternary ammonium compounds (QACs) and imidazolium ionic liquid (IIL) solvents in addition to guanidine (51, 52, 55). This suggests a broader substrate spectrum for Gdx proteins that were initially thought to have guanidine as the sole substrate (19), despite the absence of data demonstrating direct transport of QACs and ILLs. However, it is very likely that only guanidine, and its close analogs aminoguanidine and methylguanidine, induce the expression of riboswitch-controlled Gdx proteins, based on the exquisite ligand specificity previously established for guanidine riboswitches (13–16). This then raises questions on the physiological relevance of the reported Gdx-mediated resistance to QACs and ILLs that would only occur in the presence of guanidine. Notably, the addition of guanidine to the growth medium of bacteria producing biofuel was proposed as a biotechnological approach to promote Gdx-mediated resistance to ILLs contaminating the sugars used in the process that were extracted from ILL-treated biomass (55). Interestingly, *B. licheniformis* and *E. coli* strains exhibiting high tolerance to ILLs harbor mutations in their guanidine riboswitches that promote the upregulation of the ILL resistance genes they are associated with, even in the absence of guanidine (55).

In sum, the fact that several bacterial species express transporters to pump guanidine out of the cell in response to its intracellular accumulation suggests they are dealing with guanidine stress during their lifestyle. The transmission of plasmid-encoded *gdx* genes in pathogens through horizontal transfer may also be indicative of guanidine efflux being a determinant for the virulence of certain bacterial species or that these genes confer an advantage to bacteria being exposed to QACs by promoting their efflux. Again, the question of the natural source of guanidine remains. Do bacteria need to expel guanidine after it has been internally generated through metabolism or following their exposure to it in the environment, or both? The latter possibility is likely, as most riboswitch ligands, such as amino acids, RNA derivatives and precursors, signaling molecules, and polyamines, are both endogenously synthesized and imported from the environment by bacterial cells (5, 6, 35).

## BACTERIA METABOLIZE GUANIDINE

The identification of riboswitch-controlled guanidine carboxylase genes hinted at guanidine carboxylation being a probable route of guanidine assimilation, thus raising the possibility for the nitrogen-rich molecule to be used as a metabolite. Shortly after the characterization of the fourth class of guanidine riboswitch (12, 14), bacterial species using guanidine as sole nitrogen source were isolated from lake shore surface sediment in Germany (21). The enterobacteria *Raoultella terrigena*, *Erwinia rhapontici,* and *Klebsiella michiganensis* all harbor riboswitch-controlled guanidine assimilation genes, showing similar operon organization: genes encoding a guanidine importer and carboxyguanidine deaminase are associated with a guanidine-I riboswitch, while genes encoding guanidine carboxylase and allophanate hydrolase are found immediately downstream under the control of a guanidine-II riboswitch (Fig. 2). The presence of guanidine then induces a gene expression program allowing its transport and subsequent utilization. Upon guanidine import through an ABC transporter, it is converted to carboxyguanidine by guanidine carboxylase (Fig. 2). Carboxyguanidine is then transformed to allophanate through the action of carboxyguanidine deaminase, resulting in the release of ammonium. Allophanate is, in turn, hydrolyzed by allophanate hydrolase, which reaction generates two molecules of ammonia.

Two independent studies also characterized the first guanidine-degrading enzyme (GdmH) in the model cyanobacterium *Synechocystis* sp. PCC6803 (20, 56). GdmH is a highly specific Ni^2+^-dependent guanidinase from the arginase family. It was previously annotated as agmatinase, but was shown to selectively hydrolyze guanidine rather than agmatine (20). The gene encoding guanidinase is found in a guanidine-I-controlled operon together with genes encoding accessory Ni^2+^-delivery proteins required for the enzyme activity and genes encoding ABC transporters for guanidine import (Fig. 2). The presence of guanidine triggers its import and subsequent degradation into urea and ammonium by guanidinase. Urea is then converted to CO_2_ and two molecules of ammonia by urease (20). The guanidinase activity of GdmH allows *Synechocystis* sp. PCC6803 to grow with guanidine as the sole nitrogen source.

It was recently established that the complete ammonia oxidizer (comammox) *Nitrospira inopinata* can grow on guanidine as the sole source of energy, reductant, and nitrogen. This species has a GdmH-like guanidinase encoded in a riboswitch-controlled operon with an APC superfamily permease predicted to import guanidine through proton symport (57) (Fig. 2). *N. inopinata*’s guanidinase promotes guanidine hydrolysis into ammonium and urea, which is then converted to ammonia and carbon dioxide by urease (57). Ammonia released from guanidine degradation is oxidized to nitrite and then to nitrate via nitrification. While most ammonia oxidizers (organisms capable of oxidizing ammonia to nitrite, but not nitrite to nitrate) seem to rely on the guanidine carboxylase pathway, the majority of comammox species are predicted to use the guanidinase pathway for degrading guanidine (57). The latter pathway is probably more energy efficient as guanidinase does not require ATP for its activity in contrast to the ATP-dependent guanidine carboxylase. Also, guanidine transport usually occurs via ATP-dependent ABC transporters in organisms with the guanidine carboxylase pathway, while comammox are predicted to use ATP-independent APC transporters instead.

These recent findings undoubtedly provided additional insights into guanidine’s biological function, establishing that while being a stressor for some bacterial species, guanidine serves as a nitrogen source for others that evolved guanidine-specific enzymes to metabolize it. However, the question of guanidine’s natural origin remains. Are bacteria mostly exposed to external sources of guanidine, or are they endogenously producing it, or both, as it is the case for most riboswitch ligands (5)?

## LIVING ORGANISMS PRODUCE GUANIDINE

When guanidine-I riboswitch was validated (13), guanidine attracted attention as a potential biologically relevant compound that, until then, had not been considered as a metabolite. This brought up the question of whether bacteria, and potentially other living organisms, produce it. While a reporter assay using guanidine-I riboswitch as a guanidine biosensor, along with LC-MS data, suggested that guanidine is produced by *E. coli* under nutrient-poor conditions, the metabolic pathways involved in its synthesis had remained uncharacterized.

**Fig 3:**
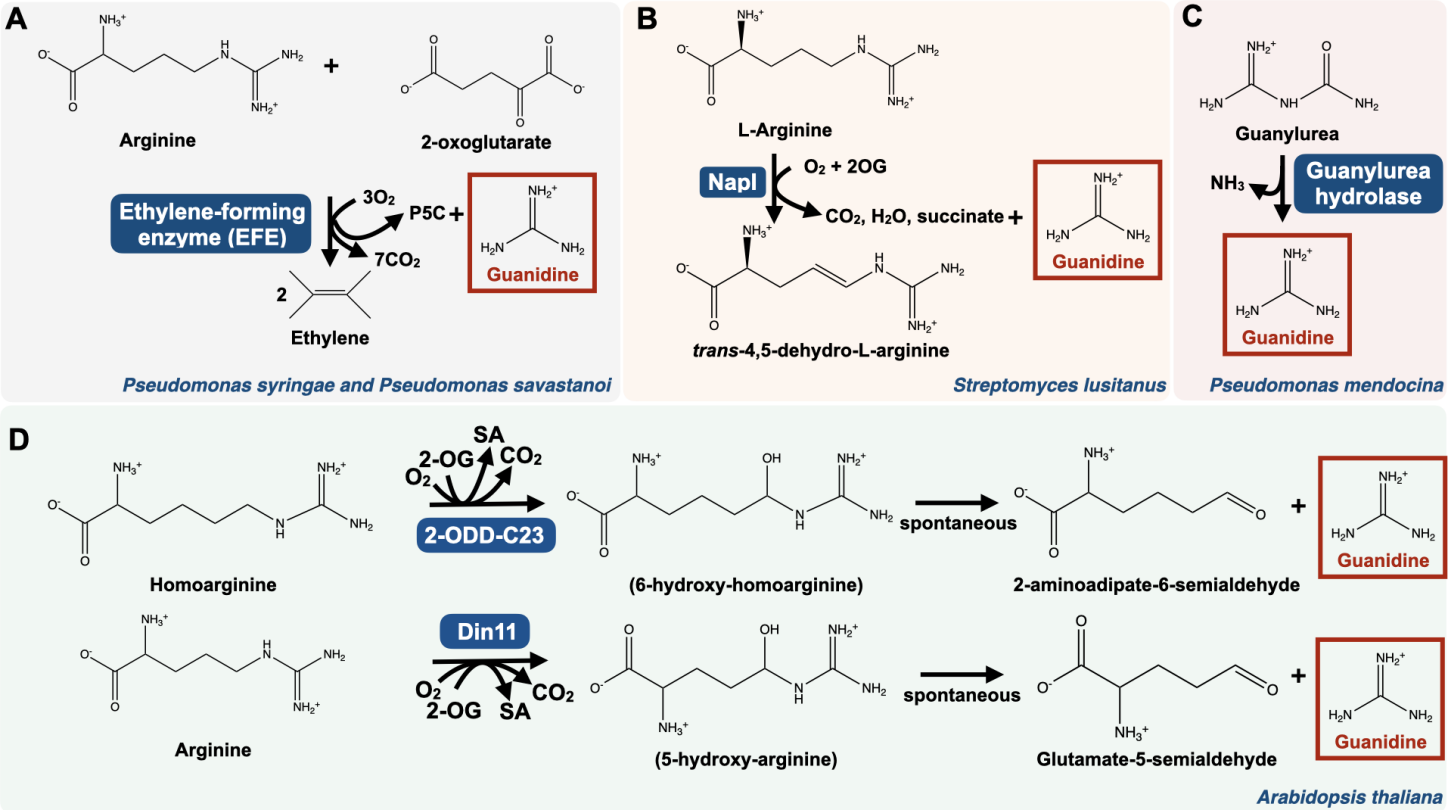
Guanidine synthesis pathways. (**A**) The ethylene-forming enzyme (EFE) pathway in *Pseudomonas syringae* and *Pseudomonas savastanoi*. P5C, pyrroline-5-carboxylate. (**B**) The NapI pathway in *Streptomyces lusitanus*. Guanidine is released as a side product of the arginine 4,5-desaturase NapI during the synthesis of the antibiotic naphthyridinomycin. (**C**) The guanylurea hydrolase pathway in *Pseudomonas mendocina*. (**D**) The homoarginine and arginine hydroxylation pathways in the plant *Arabidopsis thaliana*. 6-hydroxy-homoarginine and 5-hydroxy-arginine are too unstable to be detected. Din11 is an isoform of 2-ODD-C23 that uses arginine as substrate in addition to homoarginine. 2-ODD, 2-oxoglutarate-dependent dioxygenase; 2-OG, 2-oxoglutarate; SA, semialdehyde.

To date, three guanidine synthesis pathways are known in bacteria, and one in plants and algae. Some bacterial species, including *Pseudomonas syringae* and *Pseudomonas savastanoi*, produce guanidine from 2-oxoglutarate and arginine through the action of the ethylene-forming enzyme (EFE), which has received biotechnological interest for ethylene production (25) (Fig. 3A). In *Streptomyces lusitanus*, guanidine is released as a side product in the reaction catalyzed by the arginine-4,5-desaturase NapI during the synthesis of the antibiotic naphthyridinomycin (58) (Fig. 3B). Also, bacteria, such as *Pseudomonas mendocina*, encode guanylurea hydrolase that transforms guanylurea to ammonia and guanidine—a reaction of bioremediation interest for cleaning water from guanylurea, a compound released during the degradation of the type 2 diabetes drug metformin (23) (Fig. 3C). In plants and algae, guanidine is produced through the action of homoarginine-6-hydroxylases, which catalyze the C6-hydroxylation of homoarginine or the C5-hydroxylation of arginine, resulting in guanidine release (22) (Fig. 3D). This hints at guanidine being a relevant compound in eukaryotes, and possibly in humans, in addition to its role as a metabolite in bacteria.

These findings may take us one step forward in identifying natural sources of guanidine and their ecological relevance, with non-guanidine producers possibly being exposed to guanidine by occupying the same niches as guanidine-producing bacteria and plants.

## CONCLUDING REMARKS

The characterization of the first guanidine riboswitch nearly a decade ago (13) was a major breakthrough, motivating further investigation into the biological role of guanidine more than 150 years after the compound was first described (27). Recent studies clearly established guanidine as a metabolite that certain bacterial species use as the sole source of nitrogen (20, 21, 56), or even as a source of energy (57), and that some prokaryotic and eukaryotic organisms endogenously synthesize (22, 23, 25, 58). These findings unveiled a novel aspect of cellular metabolism and identified organisms with bioremediation potential for the removal from the environment of guanidine-rich compounds, such as the biguanidine metformin, a highly prescribed drug for treating type 2 diabetes, often found in wastewater as a contaminant (59). The presence of guanidine efflux transporters in several bacterial genomes (19) and on virulence plasmids (52) also hints at bacteria dealing with guanidine stress during their lifestyle, with the ability to respond to this insult being a possible determinant of their fitness and pathogenicity. If this is the case, guanidine efflux could constitute an attractive target for new classes of antimicrobial drugs.

Riboswitch-regulated guanidine efflux is the sole guanidine-specific stress response that has been characterized so far, with guanidine riboswitches being the only known guanidine receptors, besides guanidine transporters and guanidine-metabolizing enzymes. Future research may reveal the existence of guanidine-binding regulatory proteins, such as transcription factors or signal transduction components, triggering guanidine stress response or promoting metabolic remodeling upon guanidine sensing. Identification of environmental cues or insults leading to internal production of guanidine will also pave the way in assessing its potential role during stress response. A better understanding of the regulatory and physiological aspects of guanidine stress response will surely lead to the identification of new targets for antimicrobial drug development and provide the knowledge for genetically tweaking bacteria to enhance their resistance to guanidine stress for the purpose of bioremediation.

Despite recent advances in investigating the biological relevance of guanidine, the question of its biological source remains. As guanidine synthesis pathways have been identified in bacteria and plants (22, 23, 25, 58), one could hypothesize guanidine efflux proteins found in non-guanidine-producing bacteria to protect them against the toxic effect of guanidine synthesized by other organisms. Bacteria with the ability to grow on guanidine may also acquire the compound from external producers. Future work aiming at identifying novel guanidine biosynthesis pathways and external guanidine sources should unveil unexpected roles for guanidine in microbial communities.

One may also expect additional classes of guanidine riboswitches to be identified, further confirming the biological relevance and the possible ancient origin of the compound, with other guanidine synthesis pathways to be unveiled. Targeting these riboswitches with synthetic ligands to interfere with guanidine efflux or metabolism could be considered as a strategy of antimicrobial therapy in the future. Because of guanidine’s possible importance in plants and algae (22), regulatory pathways controlling guanidine metabolism and stress response, whose function is filled by riboswitches in bacteria, will most likely be characterized in eukaryotes.

The recent progress in elucidating the biological function of guanidine is another demonstration of the power of riboswitch validation for revealing novel aspects of bacterial physiology.
